# Measles Resurgence Associated with Continued Circulation of Genotype H1 Viruses in China, 2005

**DOI:** 10.1186/1743-422X-6-135

**Published:** 2009-09-08

**Authors:** Yixin Ji, Yan Zhang, Songtao Xu, Zhen Zhu, Shuyan Zuo, Xiaohong Jiang, Peishan Lu, Changyin Wang, Yong Liang, Huanying Zheng, Yang Liu, Naiying Mao, Xiaofeng Liang, David Alexander Featherstone, Paul A Rota, William J Bellini, Wenbo Xu

**Affiliations:** 1 WHO WPRO Regional Reference Measles Lab and State Key Laboratory for Molecular Virology & Genetic Engineering, National Institute for Viral Disease Control and Prevention, Chinese Center for Disease Control and Prevention, Beijing 100050, PR China; 2 National Immunization Program, Chinese Center for Disease Control and Prevention, Beijing, PR China; 3 Jiangsu Provincial Center for Disease Control and Prevention, PR China; 4 Shandong Provincial Center for Disease Control and Prevention, PR China; 5 Hebei Provincial Center for Disease Control and Prevention, PR China; 6 Guangdong Provincial Center for Disease Control and Prevention, PR China; 7 Tianjin Provincial Center for Disease Control and Prevention, PR China; 8 Immunization, Vaccines and Biologicals, World Health Organization, Geneva, Switzerland; 9 Division of Viral Diseases, Centers for Disease Control and Prevention, 1600 Clifton Road, Atlanta, GA 30333, USA

## Abstract

Measles morbidity and mortality decreased significantly after measles vaccine was introduced into China in 1965. From 1995 to 2004, average annual measles incidence decreased to 5.6 cases per 100,000 population following the establishment of a national two-dose regimen. Molecular characterization of wild-type measles viruses demonstrated that genotype H1 was endemic and widely distributed throughout the country in China during 1995-2004. A total of 124,865 cases and 55 deaths were reported from the National Notifiable Diseases Reporting System (NNDRS) in 2005, which represented a 69.05% increase compared with 2004. Over 16,000 serum samples obtained from 914 measles outbreaks and the measles IgM positive rate was 81%. 213 wild-type measles viruses were isolated from 18 of 31 provinces in China during 2005, and all of the isolates belonged to genotype H1. The ranges of the nucleotide sequence and predicted amino acid sequence homologies of the 213 genotype H1 strains were 93.4%-100% and 90.0%-100%, respectively. H1-associated cases and outbreaks caused the measles resurgence in China in 2005. H1 genotype has the most inner variation within genotype, it could be divided into 2 clusters, and cluster 1 viruses were predominant in China throughout 2005.

## Background

Countries in the Western Pacific Region of the World Health Organization (WHO) have identified 2012 as the target year to eliminate measles [1]. Measles continues to be a leading cause of childhood morbidity and mortality in developing countries and an outbreak threat in the majority of countries, despite the availability of an effective vaccine for 40 years [2,3].

Measles virus (MeV) is a monotype virus, but genetic variability exists among wild type strains [4]. Actually, 23 genotypes (A, B1-B3, C1-C2, D1-D10, E, F, G1-G3 and H1-H2) have been recognized circulating in different parts of the world by WHO, however five of which (B1, D1, E, F, and G1) were considered inactive since they have not been detected in the past 15 years [5,6]. Molecular epidemiologic studies can help to measure the transmission pathways of MeV and to clarify epidemiological links during outbreaks. Virological surveillance can also help to measure the success of measles vaccination programs by documenting the interruption of transmission of the endemic viral genotype(s) [7,8].

Measles morbidity and mortality decreased distinctly after measles vaccine was introduced into China in 1965. From 1995 to 2004, the average annual measles incidence decreased to 5.6 cases per 100,000 population following the establishment of a national two-dose regimen. However, the introduction of multiple outbreaks and sporadic measles cases into highly mobile communities and the accumulation of susceptible adults caused a massive spread of measles throughout China during 2005. Following standard surveillance protocols (cite WHO lab manual) serum samples and throat swabs were obtained from the suspected measles cases in 18 of 31 provinces. Measles IgM detection was used to confirm the outbreaks as being due to measles and virus isolations were performed to allow genetic characterization of the circulating strains of MeV.

## Results

### Epidemiology

**Figure 1:**
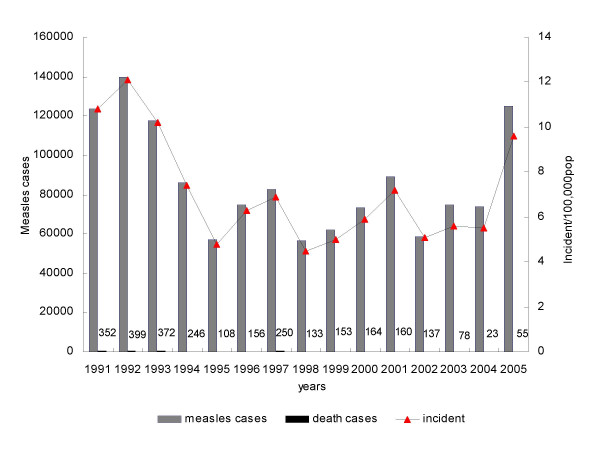
**Average number of measles cases, reported death and average measles incidence in 2005, China**. Number of reported deaths for each year is indicated above.

**Figure 2:**
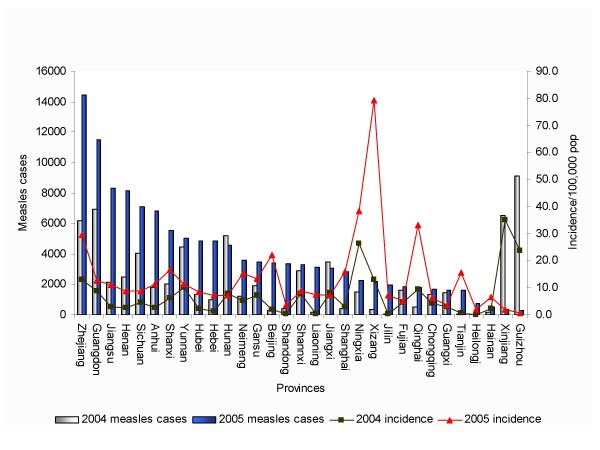
**Incidence and cases of measles in 31 provinces of China, 2004-2005**.

**Figure 3:**
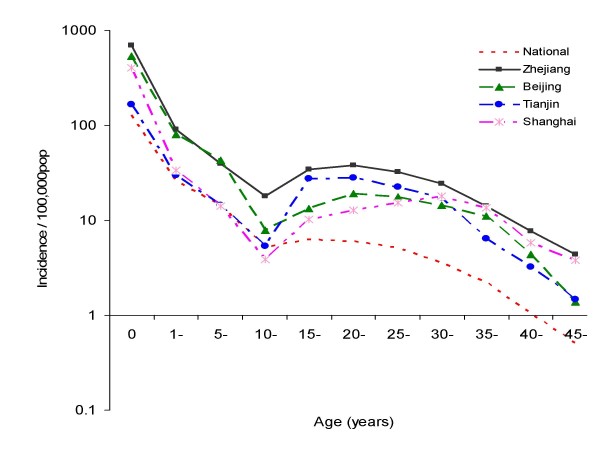
**Age-specific incidence of measles at the national level and Zhejiang, Beijing, Tianjin and Shanghai in 2005, China**.

In 2005, China experienced a large measles epidemic. A total of 124,865 cases with 55 fatalities cases were reported by NNDRS. In 14 of 31 provinces the measles incidence was >10/100,000 (Figure 1, 2). Totally, there were 914 measles outbreaks with onset in 2005 and these occurred in all 31 provinces in China. Thirty-seven percent of the cases were in the "floating populations", which are defined as persons who do not have a permanent residence card for the place in which they reside, and the proportion was over 50% in some developed provinces and big cities. The age distribution of measles changed in 2005. Compared to 2004, the measles cases in those <1 and ≥ 15 years old increased, especially in well-developed provinces such as Zhejiang, Beijing, Tianjin, Shanghai (Figure 3). Although the age-specific incidence of measles cases reported nationally was highest under 12 months, the proportion of measles cases among those aged 0-14 year-old decreased by 85% compared to 2004. In all, approximately 20% of measles cases occurred in infants under 12 months in 2005. The peak of incidence of measles occurred in the early spring.

### IgM detection

In 2005, 16,017 serum samples from 914 measles outbreaks throughout China were detected for IgM. The positive rate of measles IgM was 81% and 872 of 914 measles outbreaks were confirmed serologically.

### Molecular characterization

**Figure 4:**
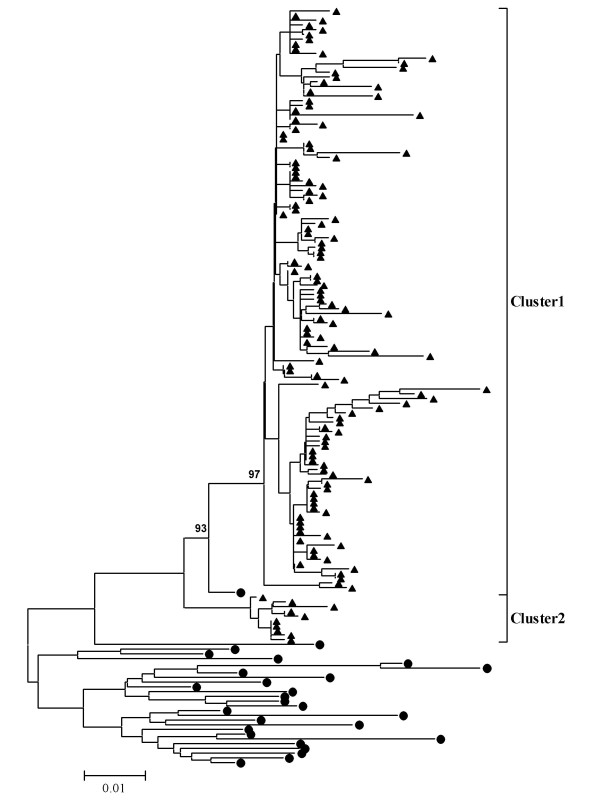
**Phylogenetic tree of measles wild-type virus strains of China in 2005 (triangles) and WHO reference MV strains (dots) based on the 456 nucleotide sequences coding for the COOH-terminus of the nucleoprotein, by using MEGA4 software and the neighbor-joining method (500 bootstraps)**. Genetic distances are represented as numbers of nucleotide differences between strains.

**Figure 5:**
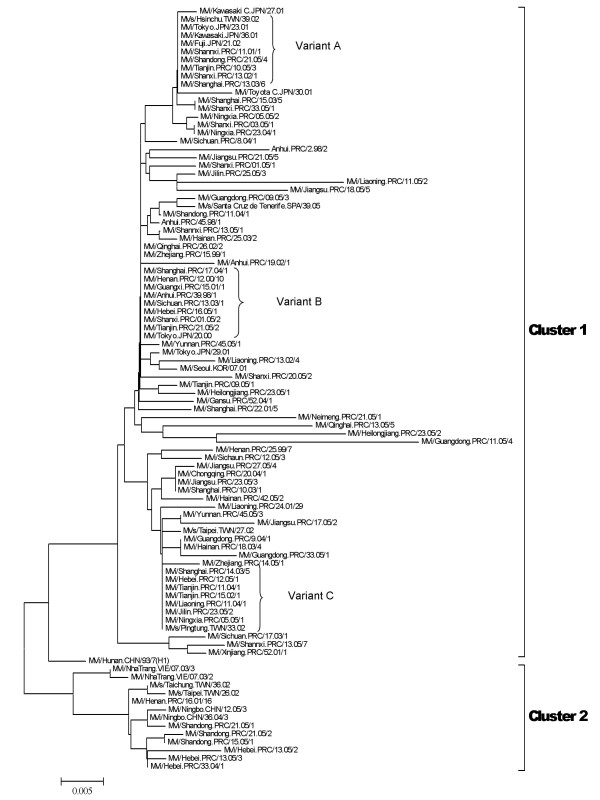
**Phylogenetic tree of Chinese representative MV strains from 1993 to 2005 and the representative MV strains of H1 genotype based on the 456 nucleotide sequences coding for the COOH-terminus of the nucleoprotein**. Three main variants (A, B and C) of cluster 1 genotype with the identical sequence for each variant were identified in mainland and Taiwan of China and Japan from 2000-2005. MeV strains of variant A were isolated from both China and Japan, 2000-2005. Variant B and variant C caused the measles continuous circulation in Mainland China and Japan from 2000-2005, and in Mainland and Taiwan China from 2002-2005, respectively. Sequences of Taiwan, South Korea, Japan and Vietnam obtained from GenBank, GenBank accession numbers are also shown for each strain.

Two hundred and thirteen wild-type measles viruses were isolated from 18 of 31 provinces in China during 2005 (Table 1). Phylogenetic analysis compared the sequences of the nucleotides coding for the carboxy terminal 150 amino acids of the N protein to the sequences of the WHO reference strains[6,9,10] (Figure 4). The clustering of MeVs in China within the genotype H1 was supported by a significant bootstrap value (500 replicates) 97% (Figure 4). Genotype H1 which was the indigenous strain in China was still the predominant circulating genotype in 2005. According to the reports about measles virus circulating in 1993-2004[11,12], H1 genotype has the greatest intra-genotype variation among all genotypes. Subsequent studies divided genotype H1 into 2 clusters, Cluster1, Cluster2 [11-15]. The phylogenetic tree showed that the sequences of genotype H1 viruses formed two major clusters (Figure 4). The amount of nucleotide variation between the two clusters was 6.6% in 2005. The phylogenetic analysis of the wild-type measles virus from China and the neighboring countries (South Korea, Japan, and Vietnam) showed that (Figure 5), with the exception of 13 sequences from Shandong, Zhejiang and Hebei provinces assigned to Cluster2, the remaining strains were members of Cluster1. Cluster1 represented the predominant lineage of endemic measles viruses in the measles outbreak in China in 2005. The Cluster2 was detected in only 3 provinces in 2005.

**Table 1:** Number of wild-type measles viruses analyzed in 2005 by province.

**Province**	**Number**	**Genotype**
Hainan	8	Cluster1
Anhui	4	Cluster1
Sichuan	5	Cluster1
Jiangsu	58	Cluster1
Ningxia	13	Cluster1
Shandong	12	Cluster1, 2
Shannxi	8	Cluster1
Zhejiang	9	Cluster1, 2*
Heilongjiang	4	Cluster1
Jilin	8	Cluster1
Hebei	21	Cluster1, 2
Guangdong	20	Cluster1
Neimeng	5	Cluster1
Qinghai	5	Cluster1
Shanxi	9	Cluster1
Yunnan	5	Cluster1
Tianjin	14	Cluster1
Liaoning	5	Cluster1

Total	213	

NOTE. -Cluster2*, including 5 isolates from Ningbo deposited in GenBank under accession numbers: DQ223905-DQ223909.

The ranges of the nucleotide sequence and predicted amino acid sequence homologies of the 213 genotype H1 strains were 93.4%-100% and 90.0%-100%, respectively. The homology of nucleotide and amino acid sequences were 84.7%-92.5% and 88.0%-92.5% respectively, when compared with that of the S191, which measles vaccine strain was used in China from 1965 to 2005.

The MV strains isolated from Japan, MVi/Tokyo.JPN/20.00 and MVi/Fuji.JPN/21.02, also have the identical sequence with Chinese endemic measles strains. Thus, the A and B variant of cluster 1(Figure 5) caused outbreak or/and sporadic cases in China during 1998-2005 and Japan in 2000 and 2002 [16-18]. Within the Cluster1 group, there was 3.3% nucleotide variation in the N gene between Chinese viruses and wiled-type measles viruses isolated in Japan and Korea during 2000 to 2002. H1 genotype, as an imported genotype, was also sporadically detected in Europe in 2005, sequence analysis confirmed suspected sources of importation [19]. In China, all major measles epidemics were associated with genotype Cluster 1 in 2005.

## Discussion

In China, measles has been classified a class B reportable disease since 1950. A two-dose measles vaccination program was introduced into China from 1986, with the first dose of vaccine administered to children at 8 months and the second dose at 7 years of age. After the vaccination plan was established, measles morbidity and mortality decreased distinctly. The current National Measles Surveillance Plan divides the provinces into national control groups. The overall incidence of measles was <8/10,000 population during 1995-2004. Provinces in Group A having an average measles incidence <6/100,000 population are developed areas and have elimination and outbreak prevention goal. Whereas, Group B (>6/100,000) provinces belong to developing areas and have a measles accelerating control goal [12].

In 2005, 5 of 31 provinces reported a measles incidence of <5/100,000. Many well-developed provinces on the east coast, that previously had low incidence rates, reported high measles incidence in 2005. The 37 percent of measles cases was happened in the floating population and the proportion was over 50% in some developed provinces and big cities. Floating people would be target group to induce measles outbreak in the cities. The age-specific incidence of measles cases was highest under 12 months and declined with increasing age from 1995-2005. However, the incidence increased in infants <1 year old, adolescents and adults >15 years old, compared to 1995 to 2004. Although the developed provinces had different incidence rates, there were no major differences in the age distribution of cases. Furthermore, the change of the measles vaccine regimen, in which the second dose was administered to children at 18-24 months from 2005, could have attributed to the change in age distribution. In 2005, there were two provinces with lower incidence, Guizhou and Xinjiang, which were performed by High-quality supplementary immunization activities (SIAs) in 2004.

Genetic analysis of wild-type measles viruses has provided an increasingly comprehensive picture of the worldwide distribution of MV genotypes [5]. In China, the initial sequencing of measles viruses was identified as a new clade H in 1998 [13,15].

In comparison with the molecular characterization of MeVs in China during 1993-2004, there was no change in genotypes in China, 2005. Gentype H1 viruses continued to circulate and were associated with imported cases in other countries [12,17,19,20]. The major neutralization sites and N-glycosylation sites in the hemagglutinin were not changed in the more recent genotype H1 viruses compared to those isolates [15]. More importantly, post-vaccination serum from individual receiving the Chinese vaccine strain, S-191, neutralized the Edmonston strain to titers that were 2-5-fold higher than the wild-type strains isolated in 1993, 1994, 1999 and 2002 (unpublished data) Therefore, the amino acid mutation in the H protein of the Chinese viruses did not appear to the result in loss of mayor neutralization epitopes by cross antibody induced following vaccination. Genetic analysis of wild-type measles isolates after the measles outbreak throughout China in 2005 indicated H1 still is the predominant genotype at present. Sequence analysis did not detect suspected sources of importation in China. Moreover, spread and prolonged circulation of similar strains has continued to cause a high number of measles cases throughout China for 13 years. As a consequence, to achieve measles elimination by 2012, measles surveillance and control need to be further optimized, and specific emphasis must be given to the vaccination of hard-to-reach populations.

## Conclusion

This study reported that the measles resurgence was caused by co-criculationg of cluster1 and cluster2 subgenotypes measles virus in China, 2005. The baseline data of virological surveillance can help to the development of improved measles control programs in China. Therefore, to eliminate measles from the Western Pacific Region by 2012, the goal set by WHO, strengthening virological surveillance capacity is crucial for monitoring the progress in measles elimination.

## Materials and methods

### Epidemiology data source

Numbers and descriptive information of measles cases and deaths in this report were from National Notifiable Diseases Reporting System of China CDC (NNDRS).

### Specimens collection and Virus isolation

Urine, throat swab and blood samples were collected from patients who had acute, febrile maculopapular rash from different provinces in China. All clinical samples were collected within five days of rash onset and transported in accordance with standard protocols[21]. Isolation of MeV was performed using the Vero/hSLAM cell line and the cells were harvested when the cytopathic effect (CPE) was visible over at least 50-75% of the cell layer[6].

### Serological testing

Commercial Enzyme-Linked Immuno Sorbent Assay (ELISA) tests were used to detect measles IgM antibody of outbreak cases through Chinese Measles Laboratory Network.

### RNA Extraction and RT-PCR

RNA was extracted from 250 μl of infected cell lysate using a Trizol reagent, followed by the manufacturer's instructions. For all virus isolates, RT-PCR amplification was performed using previously described primers to amplify a 600 bp fragment in the N gene which included the 450 bp fragment recommended for genotyping [12]. PCR products were purified using the QIAquick Gel Extraction kit (QIAGEN).

### Sequence analysis

Sequences of the PCR products were derived by automated sequencing and the BigDye terminator v2.0 chemistry according to the manufacturer's protocol in both sense and antisense strands by an automated ABI PRISM™ 3100 DNA Sequencer (Perkin Elmer), Sequence proof reading and editing was conducted with Sequencer™ (Gene Codes Corporation). Sequence data were analyzed by using version 7.0 of Bioedit and phylogenetic analyses were performed using Bioedit and Mega4 [22]. The robustness of the groupings was assessed using boorstrap resampling of 500 replicates and the trees were visualized with Mega programs. A total of 127 representative nucleotide sequences data were deposited in GenBank under accession numbers: FJ602549-FJ602674.

## List of Abbreviations

NNDRS: National Notifiable Diseases Reporting System; MeV: Measles virus; RT-PCR: reverse transcriptase polymerase chain reaction; H: Hemagglutinin; N: Nucleoprotein; WHO: World Health Organization.

## Competing interests

The authors declare that they have no competing interests.

## Authors' contributions

YXJ, WBX prepared manuscript. WBX designed the study and organized the coordination. YXJ performed RT-PCR, sequence and data analysis. YXJ, YZ, STX, ZZ, NYM performed RT-PCR and sequence analysis. XHJ, PSL, CYW, YL, HYZ, collected specimens and performed virus isolation, viral identification. All authors read and approved the final manuscript.
